# A qualitative study of how self-harm starts and continues among Chinese adolescents

**DOI:** 10.1192/bjo.2020.144

**Published:** 2020-12-17

**Authors:** Runsen Chen, Yuanyuan Wang, Li Liu, Li Lu, Amanda Wilson, Shuxiao Gong, Yingrong Zhu, Caihua Sheng, Ying Zeng, Yamin Li, Jianjun Ou

**Affiliations:** National Clinical Research Center for Mental Disorders, Department of Psychiatry, and China National Technology Institute on Mental Disorders, The Second Xiangya Hospital of Central South University, China; National Clinical Research Center for Mental Disorders, Department of Psychiatry, and China National Technology Institute on Mental Disorders, The Second Xiangya Hospital of Central South University, China; and Division of Psychology, Faculty of Health and Life Sciences, De Montfort University, UK; Department of Nursing, The Second Xiangya Hospital of Central South University, China; Bordeaux Population Health Research Center, U1219 Institut National de la Santé et de la Recherche Médicale, University of Bordeaux, France; Division of Psychology, Faculty of Health and Life Sciences, De Montfort University, UK; Department of Linguistics, University of Kansas, Kansas, USA; Division of Psychology, Faculty of Health and Life Sciences, De Montfort University, UK; Division of Psychology, Faculty of Health and Life Sciences, De Montfort University, UK; Division of Psychology, Faculty of Health and Life Sciences, De Montfort University, UK; Division of Psychology, Faculty of Health and Life Sciences, De Montfort University, UK; National Clinical Research Center for Mental Disorders, Department of Psychiatry, and China National Technology Institute on Mental Disorders, The Second Xiangya Hospital of Central South University, China

**Keywords:** Repeated self-harm, initial episode, social media, adolescent, qualitative method

## Abstract

**Background:**

It is essential to investigate the experiences behind why adolescents start and continue to self-harm in order to develop targeted treatment and prevent future self-harming behaviours.

**Aims:**

The aims of this study are to understand the motivations for initiating and repeating nonfatal self-harm, the different methods used between first-time and repeated self-harm and the reasons that adolescents do not seek help from health services.

**Methods:**

Adolescents with repeated nonfatal self-harm experiences were recruited to participate in individual, semi-structured qualitative interviews. The interviews were analysed with interpretative phenomenological analysis.

**Results:**

We found that nonfatal self-harm among adolescents occurred comparatively early and was often triggered by specific reasons. However, the subsequent nonfatal self-harm could be causeless, with repeated self-harm becoming a maladaptive coping strategy to handle daily pressure and negative emotions. The choice of tools used was related to the ease of accessibility, the life-threatening risk and the size of the scars. Adolescents often concealed their scars on purpose, which made early identification insufficient. Peer influence, such as online chat groups encouraging self-harm by discussing and sharing self-harm pictures, could also lead to increased self-harm. The results also included participants’ opinions on how to stop nonfatal self-harm and their dissatisfaction with the current healthcare services.

**Conclusions:**

The current study provides important implications both for early identification and interventions for adolescents who engage in repeated nonfatal self-harm, and for individualising treatment planning that benefits them. It is also worthwhile to further investigate how peer influence and social media may affect self-harm in adolescents.

## Background

Self-harm can be defined as any form of self-injury or self-poisoning with or without suicidal intention.^1,2^ As individuals move from childhood to adulthood, they experience various developmental transitions in their physiology, personality and sociability that could cause distress and frustration in their daily lives.^3^ Research has indicated that self-harm in adolescents is associated with multiple factors, including psychiatric difficulties and interpersonal and intrapersonal functions.^4–7^ Although the functions of self-harm remain unclear, a previous review summarised various reasons for self-harm, including alleviating negative affect and relief, self-punishment, sensation-seeking, interpersonal influence, interpersonal boundary functions and anti-suicide.^7^ Additionally, there is a widespread concern regarding the increasing trend of self-harm in adolescents and young adults.^8^ Self-harm is an important public health issue that is highly prevalent in adolescents.^9^ A recent population-based cohort study indicated that self-harm is a predictor for suicide within young people.^10^

There is a recent move to distinguish between nonfatal and fatal self-harm (e.g. self-harm with the intention of dying).^11^ The DSM-5 proposed self-harming without the intention of dying as a new diagnostic category.^12^ Nonfatal self-harm is a category of self-harm without the intention to commit suicide, and can be viewed as an expression of overwhelming psychological distress.^13^ Although the concept of nonfatal self-harm emphasises its nonsuicidal intent, the repetitiveness and severity of nonfatal self-harm are associated with a high risk of attempted suicide.^14^ In a previous study, nonfatal self-harm was a risk factor for suicidal ideation in adolescents.^15^ Additionally, individuals with a history of nonfatal self-harm during adolescence have a persistent and elevated risk of suicide attempts in adulthood,^16^ indicating that the detrimental effects of adolescent self-harm can persist as people age. Moreover, compared with infrequent self-injury, repeated self-harm leads to increased suicidal behaviours, including serious suicide attempts.^14^ During the 12-month follow-up after nonfatal self-harm, the rate of completed suicide was 439.1 per 100 000 person years, which was 37.2 times higher than the matched cohort.^17^

## Study aims

Many studies have quantitatively investigated self-harm in adolescents.^3,8,18,19^ However, there are insufficient in-depth qualitative studies focusing specifically on nonfatal self-harm in adolescents, especially repeated nonfatal self-harm. Thus, we aimed to explore and provide novel perspectives on how adolescents thought and felt about nonfatal self-harm (e.g. their self-harming experiences, self-harming methods and treatment-related issues). Through this qualitative study, we also aimed to further understand the complex nature of nonfatal self-harming in adolescents. Self-harm in adolescents could be the consequence of a mixed interaction among different factors, including biological, psychosocial and sociocultural factors. It is essential to investigate the lived experiences behind why adolescents start and continue to self-harm, to develop targeted treatment and prevent future self-harming behaviours for this population.

## Method

### Settings and participants

Adolescents with a history of nonfatal self-harm were recruited at out-patient or in-patient wards at Second Xiangya Hospital, Central South University, China. The participants met the following inclusion criteria: they were aged 10–24 years, to meet the most recent definition of adolescence;^20^ the participants could be either male or female; and they had two or more episodes of nonfatal self-harm. Participants were excluded if they were in a crisis caused by serious physical injuries resulting from self-harm, were in a state of serious illness and had difficulty communicating fluently and effectively, or exhibited a distressed emotional response when talking about self-harming. Eligible participants were referred by the hospital healthcare professionals to the research team. Participants were invited to participate via a letter sent by the members of this research team, and their attending psychiatrists were asked if the participants met the inclusion/exclusion criteria and about the current psychological well-being of the patients, to ensure that the patients were eligible to participate in the interviews. The current study was approved by the ethics committee of the Second Xiangya Hospital, Central South University, Hunan, China. All participants provided written informed consent before their enrolment (consent was obtained from the guardians if the participant was under 18 years old, and consent was received accordingly).

### Interviews and data collection

The face-to-face qualitative interviews were conducted with a semi-structured interview guide, and each lasted approximately 1 h. The interviews were conducted from August 2018 to December 2018 by two well-trained researchers who had no previous relationship with the participants. Each interview took place in a quiet interview room at the Department of Psychiatry, Central South University, China. Questions were intended to stimulate descriptions of experiences of self-harm and any perspectives they had regarding medical treatments. The initial topic guide was reviewed by qualitative experts in the mental health research field. Nonfatal self-harm was defined as self-harm without the intention to committee suicide.^13^ The nonfatal self-harm diagnosis was performed by experienced psychiatrists via hospital screening interviews. The topic guide was divided into five sections as recommended by the experts: history of self-harm and medical treatments, personal experience of self-harming behaviour, reasons for self-harming behaviour, self-harm method selection and consideration of the consequences of self-harm. The topic schedule consisted of broad and open-ended questions, such as ‘what are the factors that caused your first episode of self-harming behaviour?’ Further follow-up questions were then posed to extract details, such as ‘where did you learn this type of self-harming behaviour?’ All interviews were recorded using a digital voice recorder, and audio files were artificially transcribed into text files for subsequent analysis.

### Data analysis

The data were analysed with Interpretative Phenomenological Analysis (IPA). IPA is particularly useful for investigating the subjective nature of an individual's experience; therefore, all interviews were audio-recorded and transcribed verbatim. IPA has been used in previous qualitative studies to gain insights into how individuals make sense of their experiences when self-harming or attempting suicide. There are four phases of IPA, which include multiple readings (becoming familiar with the data) and making notes, transforming notes into emergent themes, seeking relationships and clustering the emerging themes, and producing a report.^21^ Two independent researchers separately reviewed each narrative and coded the interviews to ensure the authenticity and credibility of the results. Both researchers coded the data while data collection was occurring, to ensure that saturation was achieved. Additionally, two researchers participated in regular meetings and shared their analyses (e.g. comparing their notes) to achieve a consensus on the final themes, a common practice to account for bias during qualitative research. A qualitative expert further reviewed the themes to triangulate the data through the methodological approach of researcher triangulation. In addition, we asked the participants to review the emerging themes, to test the validity of the findings.^22^

## Results

In total, 22 participants participated in this study, and all of them completed the interview. Table 1 summarises the characteristics of each participant. There were 19 females and 3 males, with ages ranging from 12 to 24 years. Among this pool, six participants were ‘left-behind children’ (children who remained in rural regions of China, often under the care of a guardian or other relatives, while their parents left to work in urban areas), and nine participants were from single-parent families. Of those who participated, 21 participants were able to recall their first self-harm episodes. One did not provide the exact reasons for their persistent mood instability.

**Table 1:** Characteristics of each participant

Participant number	Gender	Age	Methods	Current clinical diagnosis	Trigger of first episode self-harm	Parents’ marital status	Left-behind children
1	Female	17	Knife to cut hands and arms	Social anxiety disorder/depression	Felt embarrassed when talking in front of classmates, social anxiety	Divorced	Yes
2	Female	22	Blade to cut hands and legs	Bipolar disorder	Quarrels with mother	Estranged	No
3	Female	17	Blade and compass to cut hands, and drug overdose	Depression	Quarrels with father after break-up	Living with parents	No
4	Female	21	Blade to cut hands, and drug overdose	Bipolar disorder	Persistent low mood	Living with parents	No
5	Female	15	Knife to cut hands and fingernails to cut fingers	Depression	Quarrels with mother	Estranged	No
6	Female	16	Blade to cut hands, drug overdose and hitting the wall	Depression	Hostile online messages from classmates	Living with parents	Yes
7	Female	19	Blade to cut hands, and fingernails to scratch scalp	Bipolar disorder	Quarrels with mother after truanting	Living with parents	No
8	Female	24	Blade and glass shards to cut arms and hands	Depression, eating disorder	Dissatisfaction with personal appearance and overweight	Living with parents	No
9	Female	17	Scissors to cut hands	Depression	Quarrels with boyfriend	Divorced	No
10	Male	16	Hitting the wall, slapping oneself and drug overdose	Depression	Bullied by classmates	Living with parents	No
11	Female	17	Drug overdose	Depression	Quarrels with parents because of teenage relationship	Living with parents	No
12	Female	17	Blade to cut body	Bipolar disorder	Quarrels with father because of poor academic performance	Divorced	Yes
13	Female	24	Blade to cut arm	Bipolar disorder	Quarrels with mother about sexual harassment from uncle	Divorced	No
14	Male	16	Blade to cut arms and abdomen	Depression	Quarrels with mother	Divorced	Yes
15	Female	18	Blade and compass to cut hands and thighs	Depression	Quarrels with mother	Divorced	No
16	Female	18	Hitting head against the wall and drug overdose	Depression	Depressive mood after quarrels with brother	Divorced	No
17	Female	16	Hitting head with stones	Depression	Heard quarrels between parents	Living with parents	No
18	Male	12	Slapping oneself, hitting head with glass bottle	Depression	Heard quarrels between parents	Living with parents	Yes
19	Female	20	Blade to cut arms, and drug overdose	Bipolar disorder	Quarrels with parents	Divorced	No
20	Female	19	Blade and compass to cut arm	Depression	Quarrels with parents	Living with parents	No
21	Female	21	Hitting wall with fists, and drug overdose	Bipolar disorder	Bullied and teased by classmates	Living with parents	No
22	Female	15	Glass shards to cut arms and hands	Depression	Quarrels with friends about the birth of younger brother	Divorced	Yes

We identified five themes on experiences of initiating the first self-harm episode: hopelessness derived from other people's behaviours, impulsive actions encouraged by outside instructions, relaxation and relief that resulted in regret, habitual subsequent harm that aimed to provoke intervention and wanting pleasure and pain to cope with emotions. We identified four themes on self-harm methods: choices to inflict harm, methods for causing harm and not death, inspiration to harm from media and online resources and increasing impulsivity to the point where the object of harm does not matter. Two themes emerged relating to early identification and intervention: concealment and autonomy as control mechanisms behind self-harm, and ignored help-seeking by authority figures during early episodes. One theme emerged related to the perception of treatment, which is the need for support from someone trusted for the cessation of self-harm.

### Experiences of initiating the first self-harm episode

#### Hopelessness derived from other people's behaviours

Participants reported that the reasons for the first episode of self-harm included social anxiety, interpersonal problems, dissatisfaction about one's personal appearance, relationship break-ups, being bullied by peers, relations with parents and unsatisfactory academic performance:

‘My first episode of self-harm behaviour happened when I was being bullied by my classmates. Other classmates poured ink onto me out of no reason, and then they often forced me to bark like a dog.’

‘Because my dad was so harsh on me. He said mean words to me like “With these grades, why don't you die? How could you live in this world with such disappointing grades?” And that made me feel really sad.’

#### Impulsive actions encouraged by outside instructions

The first episode was usually triggered by depressive feelings. Some participants said that the first self-harm behaviour was encouraged by friends:

‘I was furious and depressed; I didn't know what to do so I punched myself. It was beyond my control.’

‘There was no plan at all. After the big fight, I rushed to the nearby store, got a knife, and went back to my room to cut my arms. Later at night, my dad saw the scars when I was showering, and asked what happened.’

‘I asked my desk mate how to get out of a low mood. He said he was depressed as well and suggested cutting hands with a compass as a solution. I was curious so I tried it.’

#### Relaxation and relief that resulted in regret

Most participants reported that they felt relieved after self-harm, and the pain was not as strong. In addition, the pain helped them relax. However, two participants indicated that they regretted their first self-harm behaviour:

‘I felt relieved watching my blood dripping down. It was soothing.’

‘I hardly felt any pain; mostly because I was so depressed and outrageous. Hitting my head against the wall made me calm; I was not scared anymore.’

‘That day, after cutting myself, I was so regretful. I called my mom and couldn't stop crying.’

#### Habitual subsequent harm that aimed to provoke intervention

The triggers of subsequent self-harm episodes were not necessarily the same as those for the first episode, and these subsequent episodes could be provoked by minor concerns. Some participants even reported that self-harming behaviours had become a habit to deal with pressure and problems in their daily lives:

‘At first, self-harm was occasional, and gradually it grew into a habit.’

‘Self-harm is addictive, and will become more and more severe. If I accidentally died, it would be fine.’

‘No specific reason; for example, my mom always went on business trips and did not come back to see me. Once when she came home, I cut my wrist in front of her, hoping to get her attention.’

#### Wanting pleasure and pain to cope with emotions

Almost all participants considered repeated self-harm as a way of fighting against pressure or unleashing negative emotions. They could gain pleasure and relief from the repeated pain of self-harming:

‘Whenever things went a little wrong, I would harm myself.’

‘When I felt nervous in class, I just wanted to feel some pain and relax, and after cutting myself, I felt better.’

‘I once swallowed all cold and stomach medications I could find at my home. I just wanted to feel some distress.’

### Self-harm methods

#### Choices to inflict harm

Self-harm methods include self-injury (using blades, stones, compasses, or glass shards to cut oneself; punching a wall; hitting a wall with one's head; hitting one's head and slapping oneself) and self-poisoning (drug overdose, as seen above). Some participants stated that they chose these methods because they were fast, and the tools can be easily obtained:

‘When I used the scissors to cut myself every time, it would somehow relieve my emotional suffering.’

‘The compass happened to be on the table. I grabbed it and stuck it into my arm.’

‘I bought a new pair of scissors, the portable, collapsible ones. I can carry them in my pocket all the time. Whenever I felt like it, I would use them to cut myself.’

#### Methods wanted to cause harm but not death

They would also think about the life-threatening risks and continued to show that the intent was to cause harm, not to commit suicide:

‘I thought about hitting my head. But I worried it might damage my brain, or hitting my head was too noticeable. Then I chose to hit my hands using stones.’

‘I thought about climbing on a tree and jumping down, but I worried that it was too high and I might have broken my bones or died, so I did not try it.’

‘I was thinking about jumping into a car at a crossroad, but I didn't want to die from that.’

#### Inspiration to harm from media and online resources

Participants learned how to self-harm from thrillers, television shows and books. Some mentioned that they heard about it from online chat groups and even self-harmed with companions:

‘A lot of thriller movies showed that people cut their arms using knives.’

‘There is a Japanese book available online: 100 ways of self-harm.’

‘Some guys posted pictures of them doing self-harm in the chat group. They seemed cool.’

‘Sometimes I called my friends to cut ourselves together. We used knives to cut our hands.’

#### Increasing impulsivity to the point where the object of harm does not matter

Some participants mentioned that the tools for subsequent self-harm could be different from the first time, since they preferred to choose more accessible tools for subsequent self-harm:

‘I did not bring scissors to school, so I picked stones up on the playground to cut myself.’

‘From time to time I would discover more methods. They could be different from the first time. I bought a small knife for the first time. Later I lost it and did not bother to get another one.’

### Early identification and intervention

#### Concealment and autonomy as control mechanisms behind self-harm

A number of participants said that the first time they performed self-harm was a long time ago or when they were very young. There was no early intervention/identification because they did not reveal their self-harming behaviours. In addition, most of the participants mentioned that they concealed their scars on purpose, and they did not want others to know about their self-harming behaviours:

‘I don't think self-harm is a problem for myself.’

‘Why should we stop self-harm? Everyone has the right to control his or her life.’

‘I bought shirts with extra-long sleeves.’

‘I bought a watch to cover the scars.’

‘Using a knife to cut my hand was too noticeable. For better concealment, I chose to hit the wall or overdose on cold medicines.’

#### Ignored help-seeking by authority figures during early episodes

A range of participants mentioned that they did not know how to get help. The schools did not offer mental health classes and activities and remained ignorant about students’ mental health status. Although some parents and teachers may have seen their scars before, they did not pay enough attention to their self-harming behaviours:

‘I didn't take any mental health classes at school and did not know there are psychologists in our school.’

‘My parents have known about it for a long time. They just let it be.’

‘My parents punched me when they saw the scars on my hands. Then, my dad put a knife in front of me and asked if I wanted to die. This made me even more hopeless.’

‘My father never agreed to take me to the hospital. He felt ashamed to bring me to a psychiatric clinic.’

‘My roommate told my teacher about me cutting my wrist. But the teacher did not take any action.’

### Perception of treatment

#### The need for support from someone trusted for the cessation of self-harm

Participants stated that if their real problems were solved, they would no longer think about self-harm. They identified solutions to these problems such as improving family relations, getting back together with their partners or not being socially anxious anymore. They also identified that social support from parents and friends would be extremely important:

‘Medication does not help. Unless I forget this relationship and that boy, I would never feel better. Do you have a medication that can make me forget him?’

‘If I no longer feel shy and can talk to people normally, I think I will quit self-harming.’

‘Once I tried to hit the wall in my dorm. My best friend saw that and said, “If you do this again, I will, too” and I was so touched.’

In addition, some mentioned that they did not feel satisfied with healthcare services. The medical personnel did not provide enough care for them in out-patient or in-patient units. Additionally, participants mentioned that there is a lack of appropriate and effective psychological interventions for addressing self-harming behaviours:

‘Within 3 minutes of the initial assessment, the doctor asked me to be hospitalised after a very short glimpse.’

‘I did self-harming behaviour even during hospitalisation.’

‘I did not find the group therapy helpful, as I felt uncomfortable when discussing problems in front of other people.’

‘I would like to ask my mom to be involved in the psychological treatment. I believe that if she knows more about my illness, she will change her attitude towards me.’

## Discussion

This study gathered unique and specific information on how adolescents viewed nonfatal self-harm via qualitative interviews. As revealed by the findings, adolescents harmed themselves by using a range of different methods. Participants described various reasons for self-harming, and the most predominant was emotional distress (e.g. fights and arguments with family members or classmates/friends). These reasons were consistent with those given in previous studies, which found that family and school problems affected self-harm in adolescents.^23,24^ Alarmingly, participants mentioned that their self-harming behaviours were neglected, punished or considered shameful by parents or teachers.

It is critical to note the barriers of mental health help-seeking in Chinese adolescents with self-harming behaviours. There were no sufficient resources for them when needed. Participants described that they used self-harming as a way to get relief from pain and relax, indicating that nonfatal self-harming has been used as a maladaptive coping strategy for daily life distress. Andover et al stated that individuals with self-harming behaviours tended to have poor problem-solving skills and poor coping strategies.^25^ Consistent with this finding, our participants reported a lack of knowledge on dealing with emotions.

Additionally, adolescents relied on caregivers (family members, parents) and teachers to recognise their mental health issues and refer them to psychological treatments. However, participants did not receive sufficient attention and appropriate support from their parents, teachers and friends.^26^ Similar to a previous study in the UK, our participants felt patronised and ignored.^27^ Some parents of our adolescents considered self-injury to be a shameful behaviour, and these adolescents felt humiliated by their parents. Poor parenting is a risk factor for adolescent suicidal attempts in China.^28^ Thus, Chinese parents should be educated on how to interact with their child if their child is engaging in self-harming behaviours. Although the mental health problems of our participants were neglected by teachers and parents, participants’ limited access to public health services may further exacerbate these problems. Although it has been suggested that teachers should not take on the role of counsellor,^23,24^ it is important for teachers to notice students’ mental health problems and guide them to a school counsellor.

Previous research found that a friend was the main source of support for adolescents with self-harming behaviors.^29^ Consistently, our participants reported that they sought help from their friends. However, in terms of providing professional mental health support, help from a friend might play a limited role. Moreover, a friend's attitudes and behaviours toward self-harming can also negatively affect participants’ behaviours. For example, in some cases, participants adopted self-harming to cope with emotional or daily problems under the influence of their friends, a finding that was also obtained in a previous study.^24^ Additionally, friends were found to self-harm together in this study.

In the current study, some participants reported regret after self-harming. This indicates that they may not use self-harming as a solution to deal with their emotional problems if they had learned beneficial coping strategies from professionals. Early identification of adolescent self-blaming is critical to reduce the detrimental consequences for adolescent self-harm. Researchers, family members, clinicians, social workers and educational institutions should share the responsibility to ensure that the mental health of adolescents improves through the early identification of self-harming in adolescents. To promote the mental health of adolescents, Fortune et al called for effective community-based prevention and school-based programs. It is crucial for policy makers to improve service provisions to support young people experiencing self-harm.^29^ We suggest that the Chinese government should add free hotline services for adolescents with self-harming behaviours, and make psychological service units an essential infrastructure in schools. It is crucial to take adolescent self-harm seriously and to offer psychological therapies to address the distress underlying these behaviors.^30^ Screening for at-risk adolescent groups should be considered a standard procedure for self-harming prevention. For example, adolescents with depressive disorders are more likely to present with self-harming behaviors.^24^ In our clinical sample, all of the participants with self-harming behaviours suffered from mood disorders. In terms of clinicians, it is fundamental to increase the recognition of self-harm-related issues and increase compassion in therapeutic relationships.^27^

Nonfatal self-harm in adolescents has been associated with suicidal attempts in both adolescence and emerging adulthood.^15^ It is challenging to identify effective treatments for nonfatal self-harm. Prior work on interventions for self-harm in adolescents showed a paucity of evidence for effective interventions.^1^ In the current study, participants mentioned that they could stop harming themselves if their daily problems, such as lack of parental support, were solved. However, some of these problems might be difficult to change since the high proportion of left-behind children in rural areas is a widespread social phenomenon in China, caused by the large-scale immigration of the rural population to urban areas to earn a living.

Chinese left-behind children suffer from poor mental health conditions because of poor parenting and insufficient family support, posing a risk for potential nonfatal or fatal self-harming. Therapeutic interventions, including dialectical behavioural therapy, cognitive–behavioural therapy and mentalisation-based therapy, are effective at preventing self-harm in adolescents. A previous meta-analysis showed that, compared with the control group, adolescents who were in the therapeutic intervention groups had a lower proportion of self-harm (33% *v.* 28%).^31^ Moreover, recent studies have also indicated that mobile text-messaging intervention has the potential to reduce suicide and self-harm among young people who are unable to undergo face-to-face treatment.^32,33^

Our results showed that various self-harm methods were used by adolescents. It is important to note that patterns of self-harm methods could change over time. A previous meta-analysis showed that over an average of 2.8 years, one-third of participants switched methods between episodes of self-harm, with most shifting from self-injuring to self-poisoning.^34^ A suicide survey in India revealed that most people died through hanging (54%) and self-poisoning (31%).^35^ It is necessary to monitor self-harm behaviours, especially any switching of self-harm methods. The switch of self-harm methods could indicate an elevated risk of suicide. Research in the USA reported that the method choice of nonfatal self-harm episodes heightens the risk of suicide, with observations showing that persons who died by suicide applied a low-lethality method in their initial episode of self-harm, but switched to the lethal method in fatal episodes.^36^ Individuals who choose more dangerous self-harm methods should be provided with intensive follow-ups.^37^ Notably, a previous qualitative study indicated that taking away the self-harm tool could not prevent behaviours; on the contrary, it may be more harmful than helpful.^38^ Considering the elevated risk of switching self-harm methods, removing the self-harm tool could increase the risk of switching to a more lethal method. Effective preventive strategies should consider all of the potential negative consequences once the strategies are applied, and inappropriate preventive interventions could increase rather than reduce the harm.

Besides the daily problems of our participants, mood-related problems, such as depressive mood, were also found to contribute to their self-harming behaviours. We recommend treating these mood-related problems first, to decrease adolescents’ nonfatal self-harming behaviours. Supporting this idea, previous studies have shown that the incidence of self-harm caused by depression decreased after the treatment of the depression.^39^

In our sample, six participants were left-behind children, and nine were from single-parent families. It is necessary to pay specific attention to adolescents who may lack sufficient support from family members. Left-behind children suffer from severe mental health problems. For example, the prevalence of depressive symptoms among left-behind children was found to be 30.7%.^40^ A previous cross-sectional survey in China among 2898 left-behind children showed that the prevalence of self-harm was 48%. A previous survey in Belgium, using a representative school sample of 2707 adolescents, showed that boys in a single-parent family were more likely to report self-harm than boys in an intact or a remarried family, whereas girls in a remarried family were more likely to report self-harm than girls in an intact or single-parent family.^41^ Targeted interventions should be designed for adolescents from special family backgrounds, to address the insufficient emotional support and care from family members.

Our results showed that adolescents learned self-harm from television and the internet and received compassion from online groups. It is necessary to be aware of the influence of media on adolescent self-harm.^42,43^ Media depictions of self-harm could increase the risk. Recent research showed that exposure to self-harm images on Instagram was associated with later self-harm.^44^ However, considering the publicly available and explicit nature of social media, it is problematic to adequately and appropriately react in time to detrimental content on social media.^44^ A previous systematic review revealed that social media shows harmful aspects of self-harm, including normalising and accepting self-harm; however, it also shows supportive aspects, including recommendations for treatment and advice to stop self-harm and encouragement.^45^ Future studies are needed to explore the effects of media on adolescent self-harm.

This study was limited to adolescents who were willing to disclose their self-harming behaviours. It is important for future studies to investigate self-harming behaviours in a wider population, such as adolescents who do not want to disclose their problems, possibly through an anonymous online study, where this population could be more comfortable discussing their thoughts and feelings.

In conclusion, adolescent nonfatal self-harm should be taken seriously as an emerging public health issue. We should raise collaborative awareness of this serious health problem in schools, families and communities. The current findings could also assist health professionals in better recognising the complexities of the issue, and the gathered information could help in the development of future clinical interventions or therapies. There is an urgent need for adolescent nonfatal self-harming prevention programs, and public health professionals should be alerted to provide support for adolescents who engage in nonfatal self-harm.

## Data Availability

The data that support the findings of this study are available from the corresponding author, J.O., upon reasonable request.
